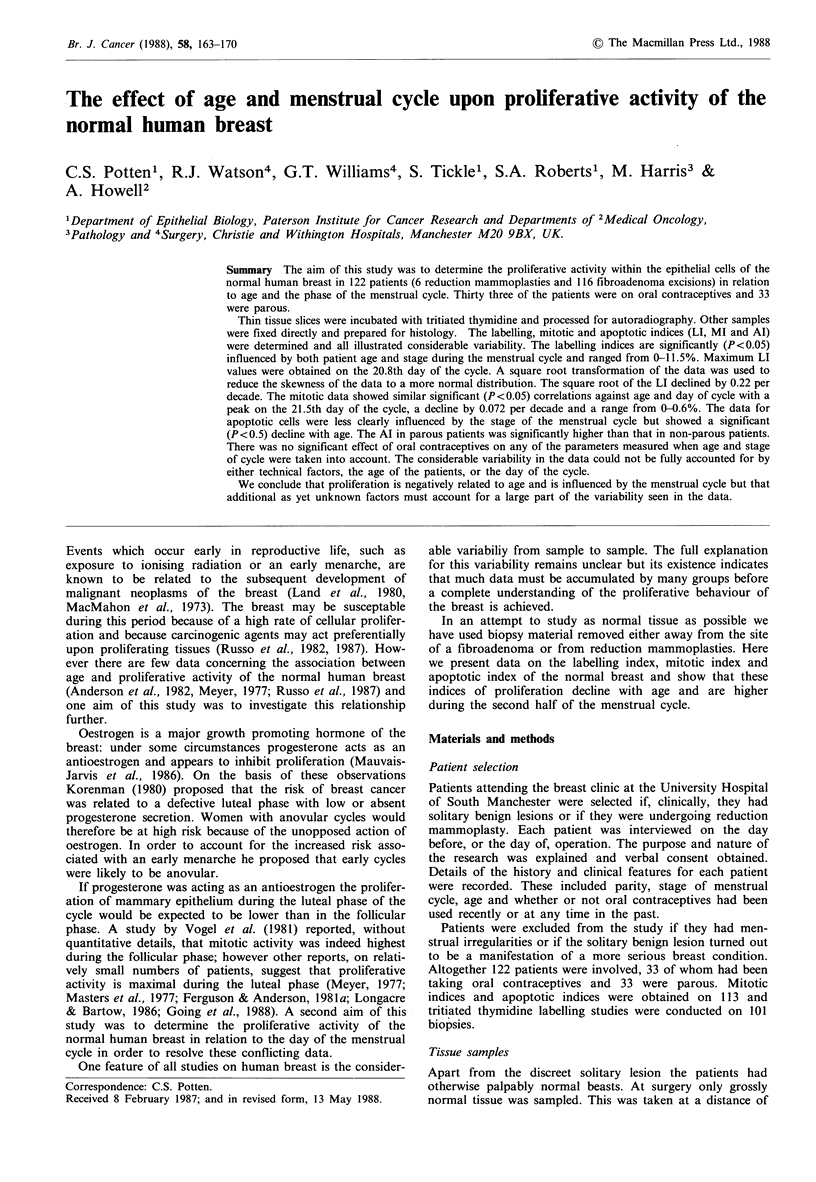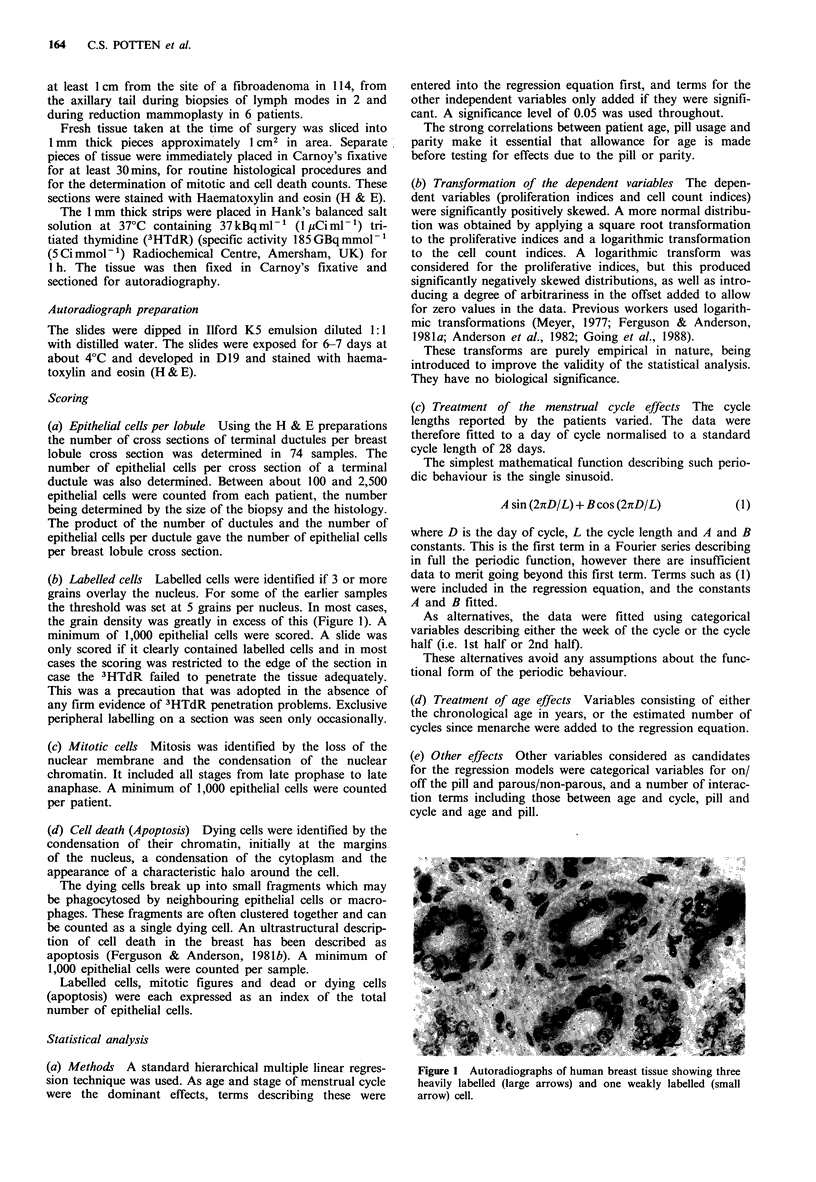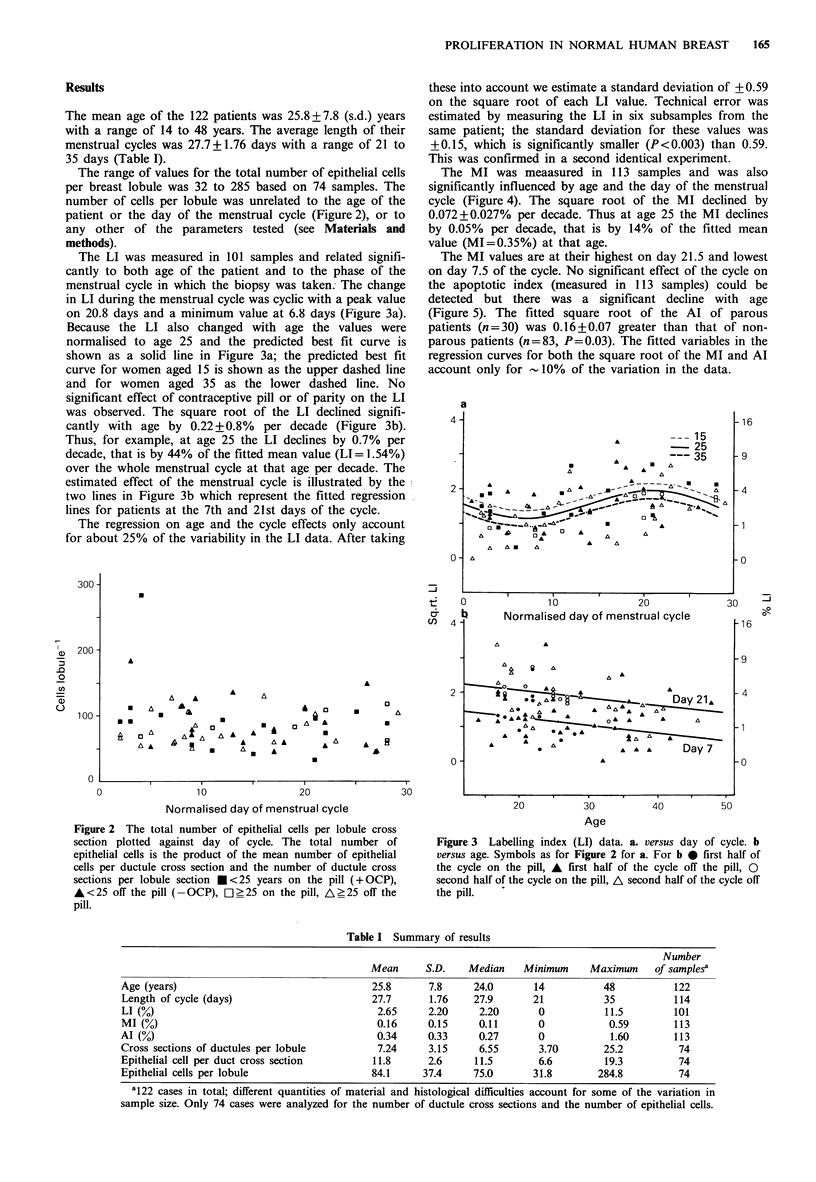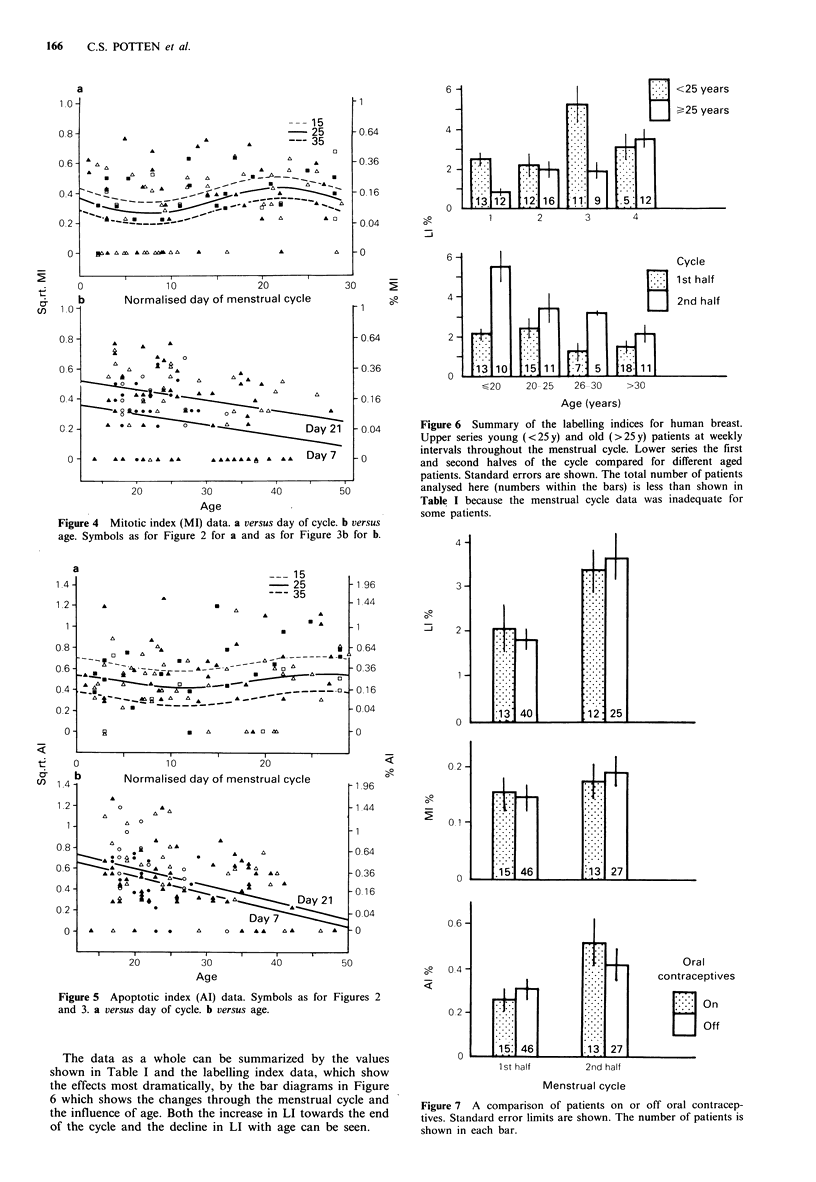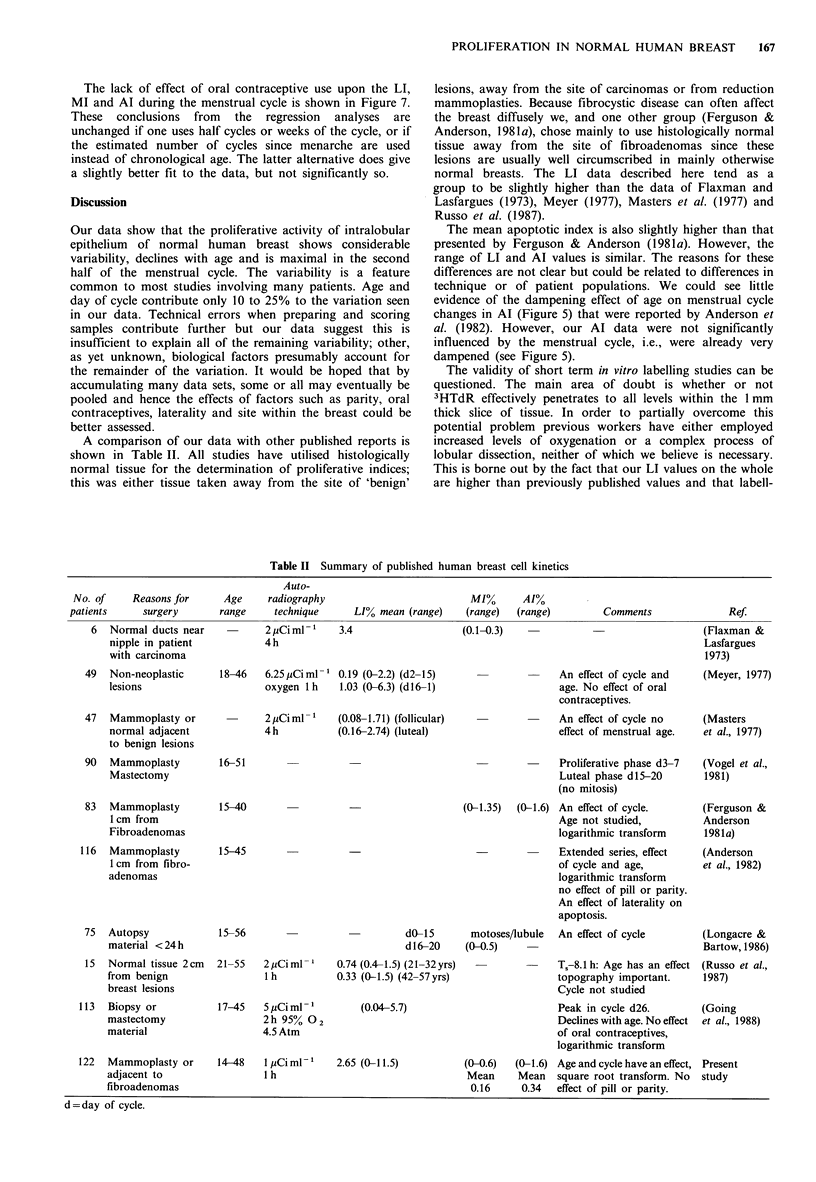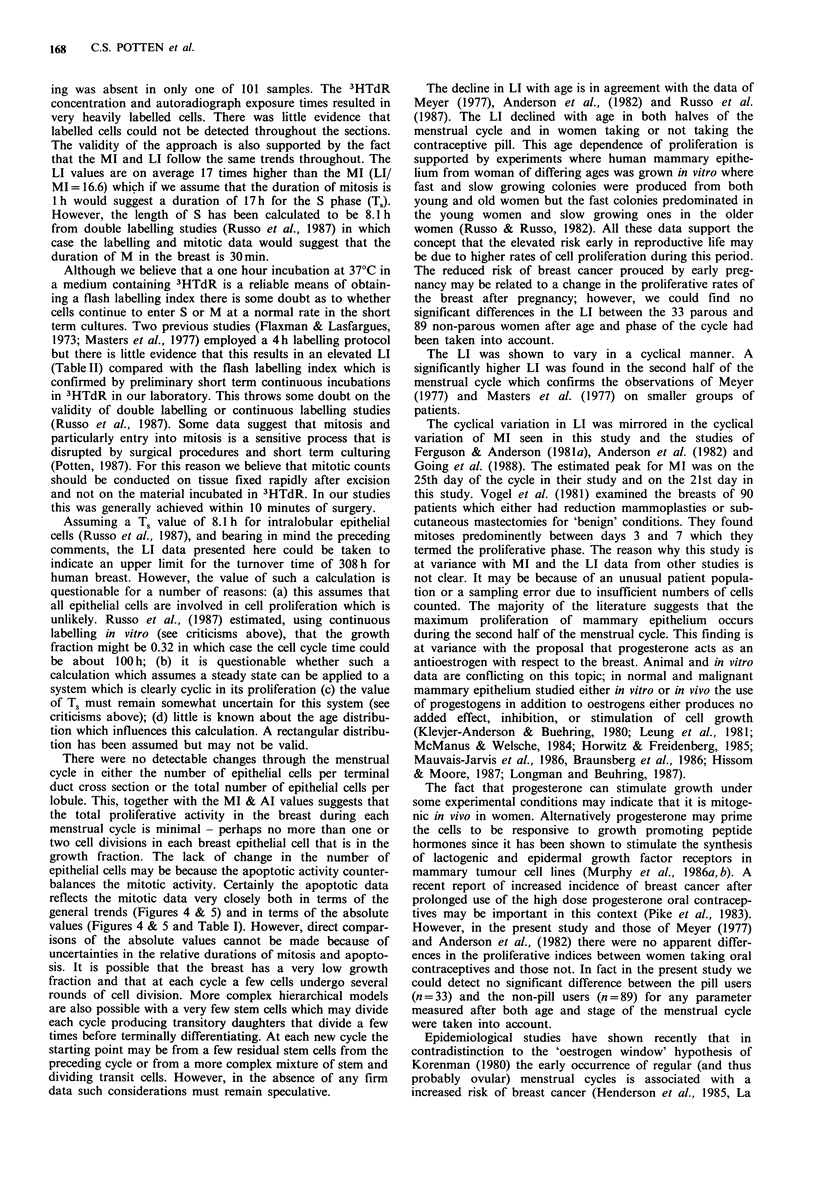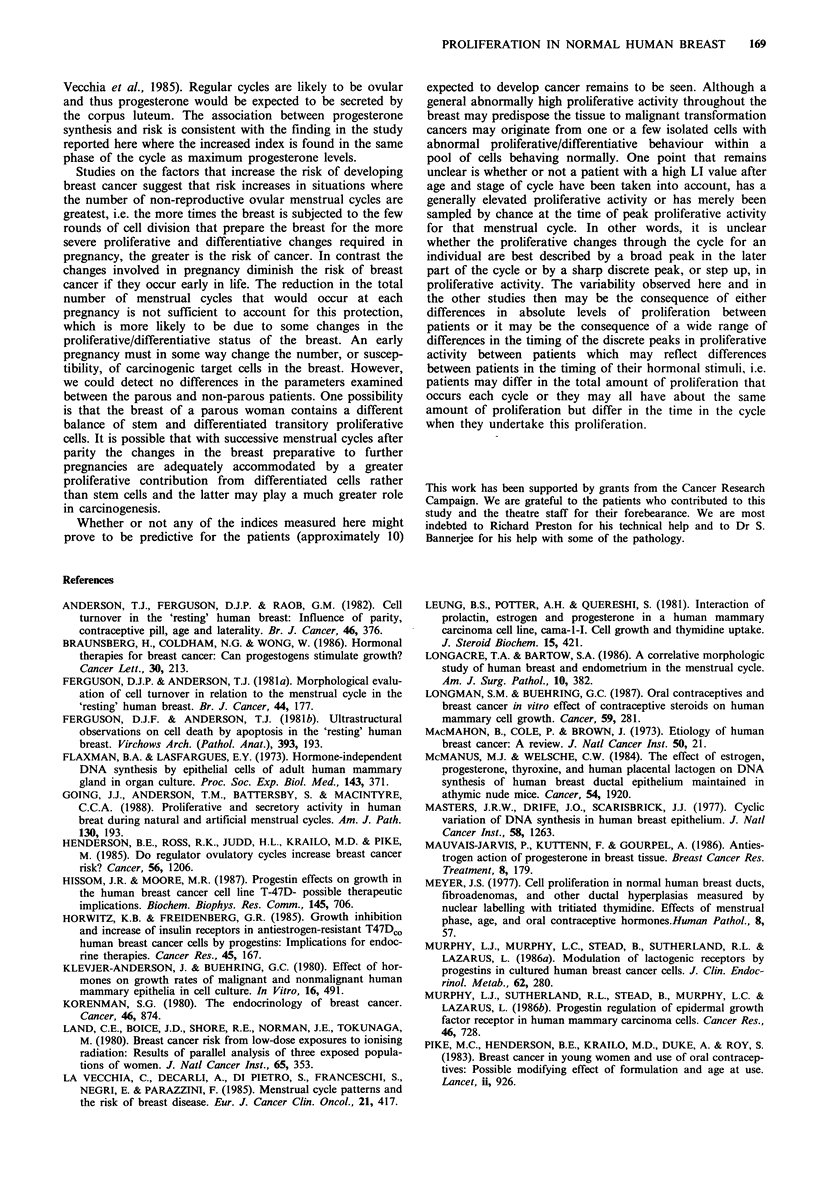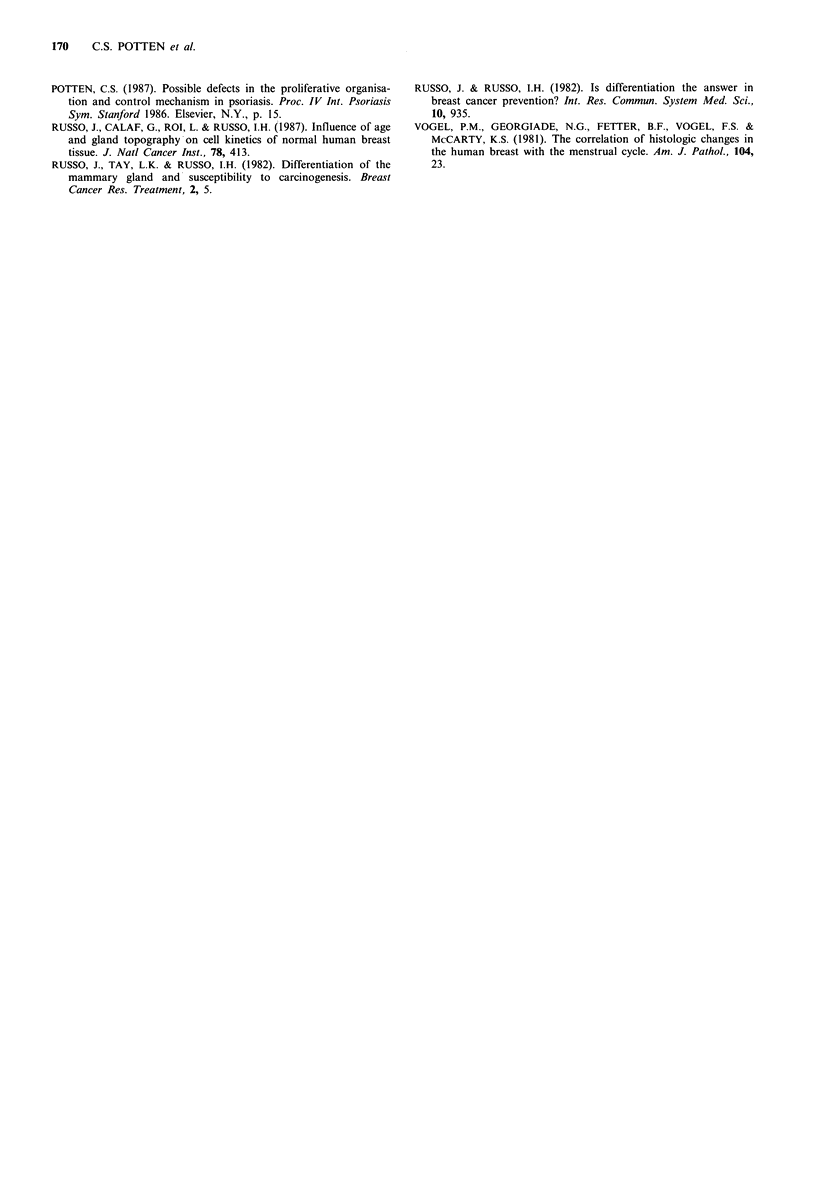# The effect of age and menstrual cycle upon proliferative activity of the normal human breast.

**DOI:** 10.1038/bjc.1988.185

**Published:** 1988-08

**Authors:** C. S. Potten, R. J. Watson, G. T. Williams, S. Tickle, S. A. Roberts, M. Harris, A. Howell

**Affiliations:** Department of Epithelial Biology, Paterson Institute for Cancer Research, Manchester, UK.

## Abstract

**Images:**


Br. J. Cancer (1988), 58, 163–170 © The Macmillan Press Ltd., 1988

### The effect of age and menstrual cycle upon proliferative activity of the normal human breast

C.S. Potten[1], R.J. Watson[4], G.T. Williams[4], S. Tickle[1], S.A. Roberts[1], M. Harris[3] & A. Howell[2]

[1]*Department of Epithelial Biology, Paterson Institute for Cancer Research and Departments of* [2]*Medical Oncology,* [3]*Pathology and* [4]*Surgery, Christie and Withington Hospitals, Manchester M20 9BX, UK.*

**Summary** The aim of this study was to determine the proliferative activity within the epithelial cells of the normal human breast in 122 patients (6 reduction mammoplasties and 116 fibroadenoma excisions) in relation to age and the phase of the menstrual cycle. Thirty three of the patients were on oral contraceptives and 33 were parous.

Thin tissue slices were incubated with tritiated thymidine and processed for autoradiography. Other samples were fixed directly and prepared for histology. The labelling, mitotic and apoptotic indices (LI, MI and AI) were determined and all illustrated considerable variability. The labelling indices are significantly ($P<0.05$) influenced by both patient age and stage during the menstrual cycle and ranged from 0–11.5%. Maximum LI values were obtained on the 20.8th day of the cycle. A square root transformation of the data was used to reduce the skewness of the data to a more normal distribution. The square root of the LI declined by 0.22 per decade. The mitotic data showed similar significant ($P<0.05$) correlations against age and day of cycle with a peak on the 21.5th day of the cycle, a decline by 0.072 per decade and a range from 0–0.6%. The data for apoptotic cells were less clearly influenced by the stage of the menstrual cycle but showed a significant ($P<0.5$) decline with age. The AI in parous patients was significantly higher than that in non-parous patients. There was no significant effect of oral contraceptives on any of the parameters measured when age and stage of cycle were taken into account. The considerable variability in the data could not be fully accounted for by either technical factors, the age of the patients, or the day of the cycle.

We conclude that proliferation is negatively related to age and is influenced by the menstrual cycle but that additional as yet unknown factors must account for a large part of the variability seen in the data.

Events which occur early in reproductive life, such as exposure to ionising radiation or an early menarche, are known to be related to the subsequent development of malignant neoplasms of the breast (Land *et al.*, 1980, MacMahon *et al.*, 1973). The breast may be susceptable during this period because of a high rate of cellular proliferation and because carcinogenic agents may act preferentially upon proliferating tissues (Russo *et al.*, 1982, 1987). However there are few data concerning the association between age and proliferative activity of the normal human breast (Anderson *et al.*, 1982, Meyer, 1977; Russo *et al.*, 1987) and one aim of this study was to investigate this relationship further.

Oestrogen is a major growth promoting hormone of the breast: under some circumstances progesterone acts as an antioestrogen and appears to inhibit proliferation (Mauvais-Jarvis *et al.*, 1986). On the basis of these observations Korenman (1980) proposed that the risk of breast cancer was related to a defective luteal phase with low or absent progesterone secretion. Women with anovular cycles would therefore be at high risk because of the unopposed action of oestrogen. In order to account for the increased risk associated with an early menarche he proposed that early cycles were likely to be anovular.

If progesterone was acting as an antioestrogen the proliferation of mammary epithelium during the luteal phase of the cycle would be expected to be lower than in the follicular phase. A study by Vogel *et al.* (1981) reported, without quantitative details, that mitotic activity was indeed highest during the follicular phase; however other reports, on relatively small numbers of patients, suggest that proliferative activity is maximal during the luteal phase (Meyer, 1977; Masters *et al.*, 1977; Ferguson & Anderson, 1981*a*; Longacre & Bartow, 1986; Going *et al.*, 1988). A second aim of this study was to determine the proliferative activity of the normal human breast in relation to the day of the menstrual cycle in order to resolve these conflicting data.

One feature of all studies on human breast is the considerable variabiliy from sample to sample. The full explanation for this variability remains unclear but its existence indicates that much data must be accumulated by many groups before a complete understanding of the proliferative behaviour of the breast is achieved.

In an attempt to study as normal tissue as possible we have used biopsy material removed either away from the site of a fibroadenoma or from reduction mammoplasties. Here we present data on the labelling index, mitotic index and apoptotic index of the normal breast and show that these indices of proliferation decline with age and are higher during the second half of the menstrual cycle.

### Materials and methods

### *Patient selection*

Patients attending the breast clinic at the University Hospital of South Manchester were selected if, clinically, they had solitary benign lesions or if they were undergoing reduction mammoplasty. Each patient was interviewed on the day before, or the day of, operation. The purpose and nature of the research was explained and verbal consent obtained. Details of the history and clinical features for each patient were recorded. These included parity, stage of menstrual cycle, age and whether or not oral contraceptives had been used recently or at any time in the past.

Patients were excluded from the study if they had menstrual irregularities or if the solitary benign lesion turned out to be a manifestation of a more serious breast condition. Altogether 122 patients were involved, 33 of whom had been taking oral contraceptives and 33 were parous. Mitotic indices and apoptotic indices were obtained on 113 and tritiated thymidine labelling studies were conducted on 101 biopsies.

### *Tissue samples*

Apart from the discreet solitary lesion the patients had otherwise palpably normal beasts. At surgery only grossly normal tissue was sampled. This was taken at a distance of

Correspondence: C.S. Potten.
Received 8 February 1987; and in revised form, 13 May 1988.

at least 1 cm from the site of a fibroadenoma in 114, from the axillary tail during biopsies of lymph modes in 2 and during reduction mammoplasty in 6 patients.

Fresh tissue taken at the time of surgery was sliced into 1 mm thick pieces approximately 1 cm$^2$ in area. Separate pieces of tissue were immediately placed in Carnoy's fixative for at least 30 mins, for routine histological procedures and for the determination of mitotic and cell death counts. These sections were stained with Haematoxylin and eosin (H & E).

The 1 mm thick strips were placed in Hank's balanced salt solution at 37°C containing 37 kBq ml$^{-1}$ (1 $\mu$Ci ml$^{-1}$) tritiated thymidine ($^3$HTdR) (specific activity 185 GBq mmol$^{-1}$ (5 Ci mmol$^{-1}$) Radiochemical Centre, Amersham, UK) for 1 h. The tissue was then fixed in Carnoy's fixative and sectioned for autoradiography.

*Autoradiograph preparation*

The slides were dipped in Ilford K5 emulsion diluted 1:1 with distilled water. The slides were exposed for 6–7 days at about 4°C and developed in D19 and stained with haematoxylin and eosin (H & E).

*Scoring*

(*a*) *Epithelial cells per lobule* Using the H & E preparations the number of cross sections of terminal ductules per breast lobule cross section was determined in 74 samples. The number of epithelial cells per cross section of a terminal ductule was also determined. Between about 100 and 2,500 epithelial cells were counted from each patient, the number being determined by the size of the biopsy and the histology. The product of the number of ductules and the number of epithelial cells per ductule gave the number of epithelial cells per breast lobule cross section.

(*b*) *Labelled cells* Labelled cells were identified if 3 or more grains overlay the nucleus. For some of the earlier samples the threshold was set at 5 grains per nucleus. In most cases, the grain density was greatly in excess of this (Figure 1). A minimum of 1,000 epithelial cells were scored. A slide was only scored if it clearly contained labelled cells and in most cases the scoring was restricted to the edge of the section in case the $^3$HTdR failed to penetrate the tissue adequately. This was a precaution that was adopted in the absence of any firm evidence of $^3$HTdR penetration problems. Exclusive peripheral labelling on a section was seen only occasionally.

(*c*) *Mitotic cells* Mitosis was identified by the loss of the nuclear membrane and the condensation of the nuclear chromatin. It included all stages from late prophase to late anaphase. A minimum of 1,000 epithelial cells were counted per patient.

(*d*) *Cell death* (*Apoptosis*) Dying cells were identified by the condensation of their chromatin, initially at the margins of the nucleus, a condensation of the cytoplasm and the appearance of a characteristic halo around the cell.

The dying cells break up into small fragments which may be phagocytosed by neighbouring epithelial cells or macrophages. These fragments are often clustered together and can be counted as a single dying cell. An ultrastructural description of cell death in the breast has been described as apoptosis (Ferguson & Anderson, 1981*b*). A minimum of 1,000 epithelial cells were counted per sample.

Labelled cells, mitotic figures and dead or dying cells (apoptosis) were each expressed as an index of the total number of epithelial cells.

*Statistical analysis*

(*a*) *Methods* A standard hierarchical multiple linear regression technique was used. As age and stage of menstrual cycle were the dominant effects, terms describing these were entered into the regression equation first, and terms for the other independent variables only added if they were significant. A significance level of 0.05 was used throughout.

The strong correlations between patient age, pill usage and parity make it essential that allowance for age is made before testing for effects due to the pill or parity.

(*b*) *Transformation of the dependent variables* The dependent variables (proliferation indices and cell count indices) were significantly positively skewed. A more normal distribution was obtained by applying a square root transformation to the proliferative indices and a logarithmic transformation to the cell count indices. A logarithmic transform was considered for the proliferative indices, but this produced significantly negatively skewed distributions, as well as introducing a degree of arbitrariness in the offset added to allow for zero values in the data. Previous workers used logarithmic transformations (Meyer, 1977; Ferguson & Anderson, 1981*a*; Anderson *et al*., 1982; Going *et al*., 1988).

These transforms are purely empirical in nature, being introduced to improve the validity of the statistical analysis. They have no biological significance.

(*c*) *Treatment of the menstrual cycle effects* The cycle lengths reported by the patients varied. The data were therefore fitted to a day of cycle normalised to a standard cycle length of 28 days.

The simplest mathematical function describing such periodic behaviour is the single sinusoid.

$$A \sin(2\pi D/L) + B \cos(2\pi D/L) \quad (1)$$

where $D$ is the day of cycle, $L$ the cycle length and $A$ and $B$ constants. This is the first term in a Fourier series describing in full the periodic function, however there are insufficient data to merit going beyond this first term. Terms such as (1) were included in the regression equation, and the constants $A$ and $B$ fitted.

As alternatives, the data were fitted using categorical variables describing either the week of the cycle or the cycle half (i.e. 1st half or 2nd half).

These alternatives avoid any assumptions about the functional form of the periodic behaviour.

(*d*) *Treatment of age effects* Variables consisting of either the chronological age in years, or the estimated number of cycles since menarche were added to the regression equation.

(*e*) *Other effects* Other variables considered as candidates for the regression models were categorical variables for on/off the pill and parous/non-parous, and a number of interaction terms including those between age and cycle, pill and cycle and age and pill.

**Figure 1** Autoradiographs of human breast tissue showing three heavily labelled (large arrows) and one weakly labelled (small arrow) cell.

**Results**

The mean age of the 122 patients was 25.8±7.8 (s.d.) years with a range of 14 to 48 years. The average length of their menstrual cycles was 27.7±1.76 days with a range of 21 to 35 days (Table I).

The range of values for the total number of epithelial cells per breast lobule was 32 to 285 based on 74 samples. The number of cells per lobule was unrelated to the age of the patient or the day of the menstrual cycle (Figure 2), or to any other of the parameters tested (see **Materials and methods**).

The LI was measured in 101 samples and related significantly to both age of the patient and to the phase of the menstrual cycle in which the biopsy was taken. The change in LI during the menstrual cycle was cyclic with a peak value on 20.8 days and a minimum value at 6.8 days (Figure 3a). Because the LI also changed with age the values were normalised to age 25 and the predicted best fit curve is shown as a solid line in Figure 3a; the predicted best fit curve for women aged 15 is shown as the upper dashed line and for women aged 35 as the lower dashed line. No significant effect of contraceptive pill or of parity on the LI was observed. The square root of the LI declined significantly with age by 0.22±0.8% per decade (Figure 3b). Thus, for example, at age 25 the LI declines by 0.7% per decade, that is by 44% of the fitted mean value (LI=1.54%) over the whole menstrual cycle at that age per decade. The estimated effect of the menstrual cycle is illustrated by the two lines in Figure 3b which represent the fitted regression lines for patients at the 7th and 21st days of the cycle.

The regression on age and the cycle effects only account for about 25% of the variability in the LI data. After taking these into account we estimate a standard deviation of ±0.59 on the square root of each LI value. Technical error was estimated by measuring the LI in six subsamples from the same patient; the standard deviation for these values was ±0.15, which is significantly smaller ($P<0.003$) than 0.59. This was confirmed in a second identical experiment.

The MI was meaasured in 113 samples and was also significantly influenced by age and the day of the menstrual cycle (Figure 4). The square root of the MI declined by 0.072±0.027% per decade. Thus at age 25 the MI declines by 0.05% per decade, that is by 14% of the fitted mean value (MI=0.35%) at that age.

The MI values are at their highest on day 21.5 and lowest on day 7.5 of the cycle. No significant effect of the cycle on the apoptotic index (measured in 113 samples) could be detected but there was a significant decline with age (Figure 5). The fitted square root of the AI of parous patients ($n=30$) was 0.16±0.07 greater than that of non-parous patients ($n=83$, $P=0.03$). The fitted variables in the regression curves for both the square root of the MI and AI account only for ~10% of the variation in the data.

**Figure 2** The total number of epithelial cells per lobule cross section plotted against day of cycle. The total number of epithelial cells is the product of the mean number of epithelial cells per ductule cross section and the number of ductule cross sections per lobule section ■<25 years on the pill (+OCP), ▲<25 off the pill (−OCP), □≧25 on the pill, △≧25 off the pill.

**Figure 3** Labelling index (LI) data. **a.** *versus* day of cycle. **b** *versus* age. Symbols as for **Figure 2** for **a**. For **b** ● first half of the cycle on the pill, ▲ first half of the cycle off the pill, ○ second half of the cycle on the pill, △ second half of the cycle off the pill.

**Table I** Summary of results

| | *Mean* | *S.D.* | *Median* | *Minimum* | *Maximum* | *Number of samples*[a] |
|---|---|---|---|---|---|---|
| Age (years) | 25.8 | 7.8 | 24.0 | 14 | 48 | 122 |
| Length of cycle (days) | 27.7 | 1.76 | 27.9 | 21 | 35 | 114 |
| LI (%) | 2.65 | 2.20 | 2.20 | 0 | 11.5 | 101 |
| MI (%) | 0.16 | 0.15 | 0.11 | 0 | 0.59 | 113 |
| AI (%) | 0.34 | 0.33 | 0.27 | 0 | 1.60 | 113 |
| Cross sections of ductules per lobule | 7.24 | 3.15 | 6.55 | 3.70 | 25.2 | 74 |
| Epithelial cell per duct cross section | 11.8 | 2.6 | 11.5 | 6.6 | 19.3 | 74 |
| Epithelial cells per lobule | 84.1 | 37.4 | 75.0 | 31.8 | 284.8 | 74 |

[a]122 cases in total; different quantities of material and histological difficulties account for some of the variation in sample size. Only 74 cases were analyzed for the number of ductule cross sections and the number of epithelial cells.

**Figure 4** Mitotic index (MI) data. **a** *versus* day of cycle. **b** *versus* age. Symbols as for Figure 2 for **a** and as for Figure 3b for **b**.

**Figure 5** Apoptotic index (AI) data. Symbols as for Figures 2 and 3. **a** *versus* day of cycle. **b** *versus* age.

The data as a whole can be summarized by the values shown in Table I and the labelling index data, which show the effects most dramatically, by the bar diagrams in Figure 6 which shows the changes through the menstrual cycle and the influence of age. Both the increase in LI towards the end of the cycle and the decline in LI with age can be seen.

**Figure 6** Summary of the labelling indices for human breast. Upper series young (<25 y) and old (>25 y) patients at weekly intervals throughout the menstrual cycle. Lower series the first and second halves of the cycle compared for different aged patients. Standard errors are shown. The total number of patients analysed here (numbers within the bars) is less than shown in Table I because the menstrual cycle data was inadequate for some patients.

**Figure 7** A comparison of patients on or off oral contraceptives. Standard error limits are shown. The number of patients is shown in each bar.

The lack of effect of oral contraceptive use upon the LI, MI and AI during the menstrual cycle is shown in Figure 7. These conclusions from the regression analyses are unchanged if one uses half cycles or weeks of the cycle, or if the estimated number of cycles since menarche are used instead of chronological age. The latter alternative does give a slightly better fit to the data, but not significantly so.

### Discussion

Our data show that the proliferative activity of intralobular epithelium of normal human breast shows considerable variability, declines with age and is maximal in the second half of the menstrual cycle. The variability is a feature common to most studies involving many patients. Age and day of cycle contribute only 10 to 25% to the variation seen in our data. Technical errors when preparing and scoring samples contribute further but our data suggest this is insufficient to explain all of the remaining variability; other, as yet unknown, biological factors presumably account for the remainder of the variation. It would be hoped that by accumulating many data sets, some or all may eventually be pooled and hence the effects of factors such as parity, oral contraceptives, laterality and site within the breast could be better assessed.

A comparison of our data with other published reports is shown in Table II. All studies have utilised histologically normal tissue for the determination of proliferative indices; this was either tissue taken away from the site of 'benign' lesions, away from the site of carcinomas or from reduction mammoplasties. Because fibrocystic disease can often affect the breast diffusely we, and one other group (Ferguson & Anderson, 1981*a*), chose mainly to use histologically normal tissue away from the site of fibroadenomas since these lesions are usually well circumscribed in mainly otherwise normal breasts. The LI data described here tend as a group to be slightly higher than the data of Flaxman and Lasfargues (1973), Meyer (1977), Masters *et al.* (1977) and Russo *et al.* (1987).

The mean apoptotic index is also slightly higher than that presented by Ferguson & Anderson (1981*a*). However, the range of LI and AI values is similar. The reasons for these differences are not clear but could be related to differences in technique or of patient populations. We could see little evidence of the dampening effect of age on menstrual cycle changes in AI (Figure 5) that were reported by Anderson *et al.* (1982). However, our AI data were not significantly influenced by the menstrual cycle, i.e., were already very dampened (see Figure 5).

The validity of short term *in vitro* labelling studies can be questioned. The main area of doubt is whether or not $^3$HTdR effectively penetrates to all levels within the 1 mm thick slice of tissue. In order to partially overcome this potential problem previous workers have either employed increased levels of oxygenation or a complex process of lobular dissection, neither of which we believe is necessary. This is borne out by the fact that our LI values on the whole are higher than previously published values and that labell-

**Table II** Summary of published human breast cell kinetics

| *No. of patients* | *Reasons for surgery* | *Age range* | *Auto-radiography technique* | *LI% mean (range)* | *MI% (range)* | *AI% (range)* | *Comments* | *Ref.* |
|---|---|---|---|---|---|---|---|---|
| 6 | Normal ducts near nipple in patient with carcinoma | — | 2 $\mu$Ci ml$^{-1}$ 4 h | 3.4 | (0.1–0.3) | — | — | (Flaxman & Lasfargues 1973) |
| 49 | Non-neoplastic lesions | 18–46 | 6.25 $\mu$Ci ml$^{-1}$ oxygen 1 h | 0.19 (0–2.2) (d2–15)<br>1.03 (0–6.3) (d16–1) | — | — | An effect of cycle and age. No effect of oral contraceptives. | (Meyer, 1977) |
| 47 | Mammoplasty or normal adjacent to benign lesions | — | 2 $\mu$Ci ml$^{-1}$ 4 h | (0.08–1.71) (follicular)<br>(0.16–2.74) (luteal) | — | — | An effect of cycle no effect of menstrual age. | (Masters *et al.*, 1977) |
| 90 | Mammoplasty Mastectomy | 16–51 | — | — | — | — | Proliferative phase d3–7 Luteal phase d15–20 (no mitosis) | (Vogel *et al.*, 1981) |
| 83 | Mammoplasty 1 cm from Fibroadenomas | 15–40 | — | — | (0–1.35) | (0–1.6) | An effect of cycle. Age not studied, logarithmic transform | (Ferguson & Anderson 1981*a*) |
| 116 | Mammoplasty 1 cm from fibro-adenomas | 15–45 | — | — | — | — | Extended series, effect of cycle and age, logarithmic transform no effect of pill or parity. An effect of laterality on apoptosis. | (Anderson *et al.*, 1982) |
| 75 | Autopsy material <24 h | 15–56 | — | — d0–15<br>d16–20 | motoses/lubule<br>(0–0.5) | —<br>— | An effect of cycle | (Longacre & Bartow, 1986) |
| 15 | Normal tissue 2 cm from benign breast lesions | 21–55 | 2 $\mu$Ci ml$^{-1}$ 1 h | 0.74 (0.4–1.5) (21–32 yrs)<br>0.33 (0–1.5) (42–57 yrs) | — | — | $T_s$–8.1 h: Age has an effect topography important. Cycle not studied | (Russo *et al.*, 1987) |
| 113 | Biopsy or mastectomy material | 17–45 | 5 $\mu$Ci ml$^{-1}$ 2 h 95% $O_2$ 4.5 Atm | (0.04–5.7) | | | Peak in cycle d26. Declines with age. No effect of oral contraceptives, logarithmic transform | (Going *et al.*, 1988) |
| 122 | Mammoplasty or adjacent to fibroadenomas | 14–48 | 1 $\mu$Ci ml$^{-1}$ 1 h | 2.65 (0–11.5) | (0–0.6) Mean 0.16 | (0–1.6) Mean 0.34 | Age and cycle have an effect, square root transform. No effect of pill or parity. | Present study |

d = day of cycle.

ing was absent in only one of 101 samples. The $^3$HTdR concentration and autoradiograph exposure times resulted in very heavily labelled cells. There was little evidence that labelled cells could not be detected throughout the sections. The validity of the approach is also supported by the fact that the MI and LI follow the same trends throughout. The LI values are on average 17 times higher than the MI (LI/MI = 16.6) which if we assume that the duration of mitosis is 1 h would suggest a duration of 17 h for the S phase ($T_s$). However, the length of S has been calculated to be 8.1 h from double labelling studies (Russo *et al.*, 1987) in which case the labelling and mitotic data would suggest that the duration of M in the breast is 30 min.

Although we believe that a one hour incubation at 37°C in a medium containing $^3$HTdR is a reliable means of obtaining a flash labelling index there is some doubt as to whether cells continue to enter S or M at a normal rate in the short term cultures. Two previous studies (Flaxman & Lasfargues, 1973; Masters *et al.*, 1977) employed a 4 h labelling protocol but there is little evidence that this results in an elevated LI (Table II) compared with the flash labelling index which is confirmed by preliminary short term continuous incubations in $^3$HTdR in our laboratory. This throws some doubt on the validity of double labelling or continuous labelling studies (Russo *et al.*, 1987). Some data suggest that mitosis and particularly entry into mitosis is a sensitive process that is disrupted by surgical procedures and short term culturing (Potten, 1987). For this reason we believe that mitotic counts should be conducted on tissue fixed rapidly after excision and not on the material incubated in $^3$HTdR. In our studies this was generally achieved within 10 minutes of surgery.

Assuming a $T_s$ value of 8.1 h for intralobular epithelial cells (Russo *et al.*, 1987), and bearing in mind the preceding comments, the LI data presented here could be taken to indicate an upper limit for the turnover time of 308 h for human breast. However, the value of such a calculation is questionable for a number of reasons: (a) this assumes that all epithelial cells are involved in cell proliferation which is unlikely. Russo *et al.*, (1987) estimated, using continuous labelling *in vitro* (see criticisms above), that the growth fraction might be 0.32 in which case the cell cycle time could be about 100 h; (b) it is questionable whether such a calculation which assumes a steady state can be applied to a system which is clearly cyclic in its proliferation (c) the value of $T_s$ must remain somewhat uncertain for this system (see criticisms above); (d) little is known about the age distribution which influences this calculation. A rectangular distribution has been assumed but may not be valid.

There were no detectable changes through the menstrual cycle in either the number of epithelial cells per terminal duct cross section or the total number of epithelial cells per lobule. This, together with the MI & AI values suggests that the total proliferative activity in the breast during each menstrual cycle is minimal – perhaps no more than one or two cell divisions in each breast epithelial cell that is in the growth fraction. The lack of change in the number of epithelial cells may be because the apoptotic activity counterbalances the mitotic activity. Certainly the apoptotic data reflects the mitotic data very closely both in terms of the general trends (Figures 4 & 5) and in terms of the absolute values (Figures 4 & 5 and Table I). However, direct comparisons of the absolute values cannot be made because of uncertainties in the relative durations of mitosis and apoptosis. It is possible that the breast has a very low growth fraction and that at each cycle a few cells undergo several rounds of cell division. More complex hierarchical models are also possible with a very few stem cells which may divide each cycle producing transitory daughters that divide a few times before terminally differentiating. At each new cycle the starting point may be from a few residual stem cells from the preceding cycle or from a more complex mixture of stem and dividing transit cells. However, in the absence of any firm data such considerations must remain speculative.

The decline in LI with age is in agreement with the data of Meyer (1977), Anderson *et al.*, (1982) and Russo *et al.* (1987). The LI declined with age in both halves of the menstrual cycle and in women taking or not taking the contraceptive pill. This age dependence of proliferation is supported by experiments where human mammary epithelium from woman of differing ages was grown *in vitro* where fast and slow growing colonies were produced from both young and old women but the fast colonies predominated in the young women and slow growing ones in the older women (Russo & Russo, 1982). All these data support the concept that the elevated risk early in reproductive life may be due to higher rates of cell proliferation during this period. The reduced risk of breast cancer prouced by early pregnancy may be related to a change in the proliferative rates of the breast after pregnancy; however, we could find no significant differences in the LI between the 33 parous and 89 non-parous women after age and phase of the cycle had been taken into account.

The LI was shown to vary in a cyclical manner. A significantly higher LI was found in the second half of the menstrual cycle which confirms the observations of Meyer (1977) and Masters *et al.* (1977) on smaller groups of patients.

The cyclical variation in LI was mirrored in the cyclical variation of MI seen in this study and the studies of Ferguson & Anderson (1981*a*), Anderson *et al.* (1982) and Going *et al.* (1988). The estimated peak for MI was on the 25th day of the cycle in their study and on the 21st day in this study. Vogel *et al.* (1981) examined the breasts of 90 patients which either had reduction mammoplasties or subcutaneous mastectomies for 'benign' conditions. They found mitoses predominently between days 3 and 7 which they termed the proliferative phase. The reason why this study is at variance with MI and the LI data from other studies is not clear. It may be because of an unusual patient population or a sampling error due to insufficient numbers of cells counted. The majority of the literature suggests that the maximum proliferation of mammary epithelium occurs during the second half of the menstrual cycle. This finding is at variance with the proposal that progesterone acts as an antioestrogen with respect to the breast. Animal and *in vitro* data are conflicting on this topic; in normal and malignant mammary epithelium studied either *in vitro* or *in vivo* the use of progestogens in addition to oestrogens either produces no added effect, inhibition, or stimulation of cell growth (Klevjer-Anderson & Buehring, 1980; Leung *et al.*, 1981; McManus & Welsche, 1984; Horwitz & Freidenberg, 1985; Mauvais-Jarvis *et al.*, 1986, Braunsberg *et al.*, 1986; Hissom & Moore, 1987; Longman and Beuhring, 1987).

The fact that progesterone can stimulate growth under some experimental conditions may indicate that it is mitogenic *in vivo* in women. Alternatively progesterone may prime the cells to be responsive to growth promoting peptide hormones since it has been shown to stimulate the synthesis of lactogenic and epidermal growth factor receptors in mammary tumour cell lines (Murphy *et al.*, 1986*a*, *b*). A recent report of increased incidence of breast cancer after prolonged use of the high dose progesterone oral contraceptives may be important in this context (Pike *et al.*, 1983). However, in the present study and those of Meyer (1977) and Anderson *et al.*, (1982) there were no apparent differences in the proliferative indices between women taking oral contraceptives and those not. In fact in the present study we could detect no significant difference between the pill users ($n = 33$) and the non-pill users ($n = 89$) for any parameter measured after both age and stage of the menstrual cycle were taken into account.

Epidemiological studies have shown recently that in contradistinction to the 'oestrogen window' hypothesis of Korenman (1980) the early occurrence of regular (and thus probably ovular) menstrual cycles is associated with a increased risk of breast cancer (Henderson *et al.*, 1985, La

Vecchia *et al.*, 1985). Regular cycles are likely to be ovular and thus progesterone would be expected to be secreted by the corpus luteum. The association between progesterone synthesis and risk is consistent with the finding in the study reported here where the increased index is found in the same phase of the cycle as maximum progesterone levels.

Studies on the factors that increase the risk of developing breast cancer suggest that risk increases in situations where the number of non-reproductive ovular menstrual cycles are greatest, i.e. the more times the breast is subjected to the few rounds of cell division that prepare the breast for the more severe proliferative and differentiative changes required in pregnancy, the greater is the risk of cancer. In contrast the changes involved in pregnancy diminish the risk of breast cancer if they occur early in life. The reduction in the total number of menstrual cycles that would occur at each pregnancy is not sufficient to account for this protection, which is more likely to be due to some changes in the proliferative/differentiative status of the breast. An early pregnancy must in some way change the number, or susceptibility, of carcinogenic target cells in the breast. However, we could detect no differences in the parameters examined between the parous and non-parous patients. One possibility is that the breast of a parous woman contains a different balance of stem and differentiated transitory proliferative cells. It is possible that with successive menstrual cycles after parity the changes in the breast preparative to further pregnancies are adequately accommodated by a greater proliferative contribution from differentiated cells rather than stem cells and the latter may play a much greater role in carcinogenesis.

Whether or not any of the indices measured here might prove to be predictive for the patients (approximately 10) expected to develop cancer remains to be seen. Although a general abnormally high proliferative activity throughout the breast may predispose the tissue to malignant transformation cancers may originate from one or a few isolated cells with abnormal proliferative/differentiative behaviour within a pool of cells behaving normally. One point that remains unclear is whether or not a patient with a high LI value after age and stage of cycle have been taken into account, has a generally elevated proliferative activity or has merely been sampled by chance at the time of peak proliferative activity for that menstrual cycle. In other words, it is unclear whether the proliferative changes through the cycle for an individual are best described by a broad peak in the later part of the cycle or by a sharp discrete peak, or step up, in proliferative activity. The variability observed here and in the other studies then may be the consequence of either differences in absolute levels of proliferation between patients or it may be the consequence of a wide range of differences in the timing of the discrete peaks in proliferative activity between patients which may reflect differences between patients in the timing of their hormonal stimuli, i.e. patients may differ in the total amount of proliferation that occurs each cycle or they may all have about the same amount of proliferation but differ in the time in the cycle when they undertake this proliferation.

This work has been supported by grants from the Cancer Research Campaign. We are grateful to the patients who contributed to this study and the theatre staff for their forebearance. We are most indebted to Richard Preston for his technical help and to Dr S. Bannerjee for his help with some of the pathology.

### References

ANDERSON, T.J., FERGUSON, D.J.P. & RAOB, G.M. (1982). Cell turnover in the 'resting' human breast: Influence of parity, contraceptive pill, age and laterality. *Br. J. Cancer*, **46**, 376.

BRAUNSBERG, H., COLDHAM, N.G. & WONG, W. (1986). Hormonal therapies for breast cancer: Can progestogens stimulate growth? *Cancer Lett.*, **30**, 213.

FERGUSON, D.J.P. & ANDERSON, T.J. (1981*a*). Morphological evaluation of cell turnover in relation to the menstrual cycle in the 'resting' human breast. *Br. J. Cancer*, **44**, 177.

FERGUSON, D.J.F. & ANDERSON, T.J. (1981*b*). Ultrastructural observations on cell death by apoptosis in the 'resting' human breast. *Virchows Arch.* (*Pathol. Anat.*), **393**, 193.

FLAXMAN, B.A. & LASFARGUES, E.Y. (1973). Hormone-independent DNA synthesis by epithelial cells of adult human mammary gland in organ culture. *Proc. Soc. Exp. Biol. Med.*, **143**, 371.

GOING, J.J., ANDERSON, T.M., BATTERSBY, S. & MACINTYRE, C.C.A. (1988). Proliferative and secretory activity in human breat during natural and artificial menstrual cycles. *Am. J. Path.* **130**, 193.

HENDERSON, B.E., ROSS, R.K., JUDD, H.L., KRAILO, M.D. & PIKE, M. (1985). Do regulator ovulatory cycles increase breast cancer risk? *Cancer*, **56**, 1206.

HISSOM, J.R. & MOORE, M.R. (1987). Progestin effects on growth in the human breast cancer cell line T-47D- possible therapeutic implications. *Biochem. Biophys. Res. Comm.*, **145**, 706.

HORWITZ, K.B. & FREIDENBERG, G.R. (1985). Growth inhibition and increase of insulin receptors in antiestrogen-resistant $T47D_{co}$ human breast cancer cells by progestins: Implications for endocrine therapies. *Cancer Res.*, **45**, 167.

KLEVJER-ANDERSON, J. & BUEHRING, G.C. (1980). Effect of hormones on growth rates of malignant and nonmalignant human mammary epithelia in cell culture. *In Vitro*, **16**, 491.

KORENMAN, S.G. (1980). The endocrinology of breast cancer. *Cancer*, **46**, 874.

LAND, C.E., BOICE, J.D., SHORE, R.E., NORMAN, J.E., TOKUNAGA, M. (1980). Breast cancer risk from low-dose exposures to ionising radiation: Results of parallel analysis of three exposed populations of women. *J. Natl Cancer Inst.*, **65**, 353.

LA VECCHIA, C., DECARLI, A., DI PIETRO, S., FRANCESCHI, S., NEGRI, E. & PARAZZINI, F. (1985). Menstrual cycle patterns and the risk of breast disease. *Eur. J. Cancer Clin. Oncol.*, **21**, 417.

LEUNG, B.S., POTTER, A.H. & QUERESHI, S. (1981). Interaction of prolactin, estrogen and progesterone in a human mammary carcinoma cell line, cama-1-I. Cell growth and thymidine uptake. *J. Steroid Biochem.* **15**, 421.

LONGACRE, T.A. & BARTOW, S.A. (1986). A correlative morphologic study of human breast and endometrium in the menstrual cycle. *Am. J. Surg. Pathol.*, **10**, 382.

LONGMAN, S.M. & BUEHRING, G.C. (1987). Oral contraceptives and breast cancer *in vitro* effect of contraceptive steroids on human mammary cell growth. *Cancer*, **59**, 281.

MACMAHON, B., COLE, P. & BROWN, J. (1973). Etiology of human breast cancer: A review. *J. Natl Cancer Inst.* **50**, 21.

MCMANUS, M.J. & WELSCHE, C.W. (1984). The effect of estrogen, progesterone, thyroxine, and human placental lactogen on DNA synthesis of human breast ductal epithelium maintained in athymic nude mice. *Cancer*, **54**, 1920.

MASTERS, J.R.W., DRIFE, J.O., SCARISBRICK, J.J. (1977). Cyclic variation of DNA synthesis in human breast epithelium. *J. Natl Cancer Inst.*, **58**, 1263.

MAUVAIS-JARVIS, P., KUTTENN, F. & GOURPEL, A. (1986). Antiestrogen action of progesterone in breast tissue. *Breast Cancer Res. Treatment*, **8**, 179.

MEYER, J.S. (1977). Cell proliferation in normal human breast ducts, fibroadenomas, and other ductal hyperplasias measured by nuclear labelling with tritiated thymidine. Effects of menstrual phase, age, and oral contraceptive hormones.*Human Pathol.*, **8**, 57.

MURPHY, L.J., MURPHY, L.C., STEAD, B., SUTHERLAND, R.L. & LAZARUS, L. (1986*a*). Modulation of lactogenic receptors by progestins in cultured human breast cancer cells. *J. Clin. Endocrinol. Metab.*, **62**, 280.

MURPHY, L.J., SUTHERLAND, R.L., STEAD, B., MURPHY, L.C. & LAZARUS, L. (1986*b*). Progestin regulation of epidermal growth factor receptor in human mammary carcinoma cells. *Cancer Res.*, **46**, 728.

PIKE, M.C., HENDERSON, B.E., KRAILO, M.D., DUKE, A. & ROY, S. (1983). Breast cancer in young women and use of oral contraceptives: Possible modifying effect of formulation and age at use. *Lancet*, **ii**, 926.

POTTEN, C.S. (1987). Possible defects in the proliferative organisation and control mechanism in psoriasis. *Proc. IV Int. Psoriasis Sym. Stanford* 1986. Elsevier, N.Y., p. 15.

RUSSO, J., CALAF, G., ROI, L. & RUSSO, I.H. (1987). Influence of age and gland topography on cell kinetics of normal human breast tissue. *J. Natl Cancer Inst.*, **78**, 413.

RUSSO, J., TAY, L.K. & RUSSO, I.H. (1982). Differentiation of the mammary gland and susceptibility to carcinogenesis. *Breast Cancer Res. Treatment*, **2**, 5.

RUSSO, J. & RUSSO, I.H. (1982). Is differentiation the answer in breast cancer prevention? *Int. Res. Commun. System Med. Sci.*, **10**, 935.

VOGEL, P.M., GEORGIADE, N.G., FETTER, B.F., VOGEL, F.S. & McCARTY, K.S. (1981). The correlation of histologic changes in the human breast with the menstrual cycle. *Am. J. Pathol.*, **104**, 23.